# Two new endemic tree species from Puerto Rico: *Pisonia horneae* and *Pisonia roqueae* (Nyctaginaceae)

**DOI:** 10.3897/phytokeys.86.11249

**Published:** 2017-09-26

**Authors:** Marcos A. Caraballo-Ortiz, Jorge C. Trejo-Torres

**Affiliations:** 1 The Pennsylvania State University, Department of Biology, 208 Mueller Laboratory, University Park, Pennsylvania, 16802, USA; 2 The Institute for Regional Conservation, 100 East Linton Boulevard, Suite 302B, Delray Beach, Florida, 33483, USA

**Keywords:** Ana Roqué de Duprey, Caribbean, Caryophyllales, Frances W. Horne, *Pisonia subcordata* var. *typica* f. *gigantophylla*, “*Pisonia borinquena*”, “*Pisonia woodburyana*”, West Indies

## Abstract

In this paper, we describe two endemic tree species of *Pisonia* (Caryophyllales: Nyctaginaceae) from Puerto Rico that were erroneously catalogued under the single name Pisonia subcordata var. typica f. gigantophylla, misidentified as *P. albida* or *P. subcordata*, and informally named as “*P. borinquena*” and “*P. woodburyana*”. The species here named as *P. horneae* is a rare to locally occasional tree from low elevations in the Northern Karst and the Sierra de Cayey. The other species, here named as *P. roqueae*, is a rare to locally common tree from mid to high elevations in the Central Mountain Range and the Luquillo Mountains. We provide an account of the taxonomical and nomenclatural history of both species, images, conservation notes, a distribution map, and a key to distinguish the species of *Pisonia* present in Puerto Rico.

## Abstract (es)

En esta publicación se describen dos especies de árboles endémicos de *Pisonia* (Caryophyllales: Nyctaginaceae) de Puerto Rico, las cuales fueron erróneamente catalogadas bajo un solo nombre como Pisonia subcordata var. typica f. gigantophylla, mal identificadas como *P. albida* o *P. subcordata*, e informalmente nombradas como “*P. borinquena*” y “*P. woodburyana*”. La especie aquí nombrada como *P. horneae* es un árbol raro a localmente ocasional de bajas elevaciones en el Karso Norteño y la Sierra de Cayey. La segunda especie aquí nombrada como *P. roqueae* es un árbol raro o localmente común de elevaciones medias a altas en la Cordillera Central y las Montañas de Luquillo. Se provee un recuento de la historia taxonómica y nomenclatural de ambas especies, imágenes, notas de conservación, un mapa de distribución, y una clave para distinguir las especies de *Pisonia* presentes en Puerto Rico.

## Introduction

The Caribbean Islands are considered one of the hottest hotspots of world biodiversity (Myers et al. 2000) with a richness that has not been fully documented yet, as evidenced by the dozens of new species described from the region every year (Hedges and Conn 2012, Corgosinho and Schizas 2013, Mercado-Diaz et al. 2014, Cardoso and Braga 2015, Aguirre-Santoro et al. 2016, Carmenate-Reyes et al. 2017). For example, Puerto Rico – the smallest of the Greater Antilles – is probably the island with the best documented flora of the region with about 2331 species of native plants (Gann et al. 2015-2017). In spite of the intensive botanical work conducted in the island for more than a century, new species and rediscoveries of plants are still being documented (e.g., in the last decade: Trejo-Torres 2008, 2009, Vega et al. 2010, Caraballo-Ortiz 2013, Bornstein et al. 2014, Trejo-Torres et al. 2014).

### An uncertain name used for a *Pisonia*

Pisonia subcordata Swartz var. typica Heimerl f. gigantophylla Heimerl (Caryophyllales: Nyctaginaceae) was described by Heimerl (1896) using a set of sterile specimens gathered by Paul E. E. Sintenis from at least two localities in central Puerto Rico. Nine years later, Heimerl and Urban (1905) synonymized this name under Pisonia subcordata var. typica Heimerl, as they considered the type collections to be juvenile parts of this species. In 1918, Standley reclassified P. subcordata var. typica f. gigantophylla as a synonym of *P. albida* (Heimerl) Britton *ex* Standley (Standley 1918). Coincidently, in 1980 Westra examined and annotated the duplicates of specimen Sintenis 2705 (B, K) and considered them as a mixed collection containing parts of *P. albida* and *P. subcordata* Swartz.

After examining the type material of the abovementioned taxa, additional specimens, and living plants, we have concluded that the three sets of specimens used to describe P. subcordata var. typica f. gigantophylla (Sintenis 2141, 2705, and 4355 [sic. 4335]) comprise two taxa, which however, do not match any of the currently recognized *Pisonia* species from Puerto Rico or the Caribbean. While the two duplicates of Sintenis 2141 (GH, K) represent the same taxon, at least two of the three duplicates of Sintenis 2705 (B, K, and possibly GH) are mixed and include the same taxon as Sintenis 2141 as well as a second taxon. Although these specimens clearly represent two taxa, their sterile and fragmented nature, as well as the scant protologue, brings uncertainties to assign identities to each of the pieces. Moreover, Sintenis 4355 has not been located yet, and we lack information on its identity or locality.

### Two informal names for *Pisonia*

In 1985 and 1993, George R. Proctor informally recognized two species of *Pisonia* from Puerto Rico and annotated a series of specimens as “*Pisonia borinquena* Proctor” (Britton 5628 (NY); Caminero 282, 443 (MAPR); Little 13380, 13404 (NY); Proctor 45465 (FTG, UPR); Proctor 43322, 44432, 48064 (US)) and “*Pisonia woodburyana* Proctor” (Britton 1515 (NY); Gleason A134 (NY); Liogier 10670 (NY, US); Proctor 41173 (IJ, UPR); Proctor 43284, 45450 (US); Woodbury s.n. (NY)). These unpublished names have been used in other herbarium specimens and databases, as well as in floristic assessments, scientific presentations and publications, and technical reports (e.g., Francis et al. 1998, Acevedo-Rodríguez and Axelrod 1999, Antoun et al. 1999, 2001, Alemañy-Merly 2000, Caraballo-Ortiz and Santiago-Valentín 2005, Caraballo-Ortiz 2007, Axelrod 2011, Trejo-Torres et al. 2011, Gann et al. 2015–2017).

Our analysis of living and dried specimens shows that the two species of *Pisonia* recognized by Proctor represent the same two taxa involved in the description of P. subcordata var. typica f. gigantophylla. Therefore, our objective in this paper is to clarify the identities and assign new names for these two species of *Pisonia*. For each species, we provide descriptions, illustrations, and comments on their abundance, natural history and conservation. In addition, we provide a distribution map and a dichotomous artificial key for the six species of *Pisonia* reported for Puerto Rico.

## Materials and methods

Information on living plants and natural history was obtained from field observations and cultivated plants growing at the nursery of the Conservation Trust of Puerto Rico at San Juan. Species descriptions and measurements were obtained from field notes and dried herbarium specimens.

## Taxonomic treatment

### Pisonia horneae

Taxon classificationPlantaeCaryophyllalesNyctaginaceae

Trejo & Caraballo sp. nov.

urn:lsid:ipni.org:names:77165902-1

Figs 1 , 2 , 3 , 4

Pisonia subcordata sensu Heimerl and Urban (1905), not Swartz (1788).
Pisonia subcordata Swartz var. typica Heimerl f. gigantophylla Heimerl, Bot. Jahrb. Syst. 21: 630. 1896. Pro parte (see Additional specimens examined section).
“Pisonia woodburyana Proctor”, *nomen nudum*.

#### Type.

PUERTO RICO. Municipality of Quebradillas, Bo. Terranova, old train road to El Tunel Negro, from Highway #2, border of dirt road near GPS [18.4840°, -66.9536°], 5 m, 12 Jul 2003, J.C. Trejo, P. Vives, A.M. Camacho, and M.A. Caraballo 2310 (holotype: UPR!; isotypes: FLAS! [#230768, 230769]).

#### Diagnosis.

*Pisonia horneae* is distinguished from congeners from Puerto Rico by a combination of the following characters: leaves membranaceous and puberulous, twigs puberulous, fruits elliptic-oblanceolate and grayish with 10 rows of viscid glands along their whole length.

**Figure 1.**
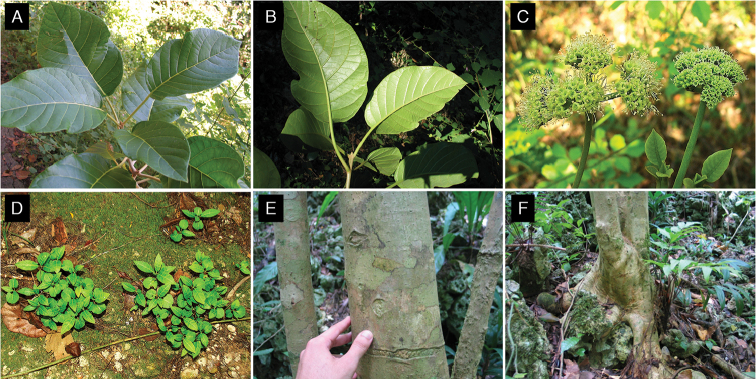
*Pisonia horneae*. **A** Branch showing adaxial side of leaves **B** Abaxial side of leaves **C** Staminate (left) and pistilate (right) flowers at anthesis **D** Seedlings **E** Bark **F** Lower trunk of an adult tree. Note the characteristic swollen base observed in all *Pisonia* from Puerto Rico, where the trunk base wraps over the rocks as it grows. Photo credits: **A–B, E–F**: JCTT; **C–D**: MACO.

**Figure 2.**
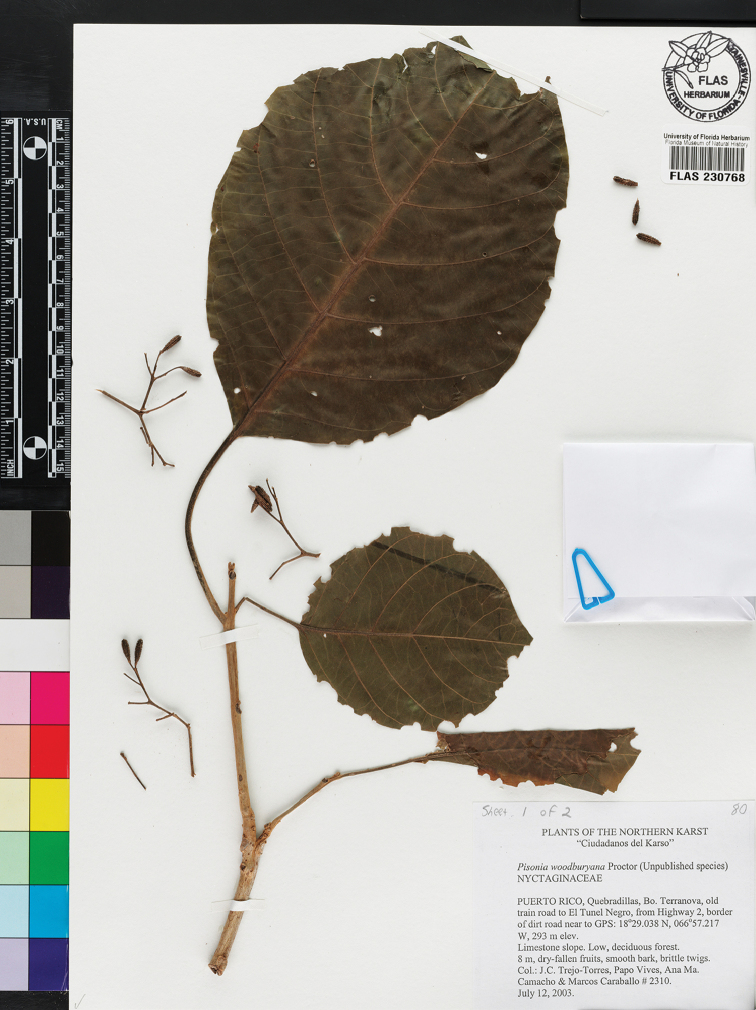
Isotype specimen of *Pisonia horneae* from the University of Florida Herbarium (Trejo et al. 2310, FLAS [230768]). Photo courtesy of The University of Florida Herbarium (FLAS) – Florida Museum of Natural History.

#### Description.

***Trees*** dioecious to 10 m high with trunks to 30 cm in diameter. ***Bark*** finely and vertically striated, hazel, grayish, or silvery, sometimes lustrous, with lenticels about 3 mm in diameter. ***Twigs*** slightly ancipitous, greenish, and puberulous when young; terete, puberulent, and grayish when old. ***Leaves*** deciduous, clustered towards the ends of branches; opposite or subopposite, decussate; petioles to 7 cm long, grayish; blades ovate, elliptic, obovate, or roundish, sometimes asymmetric, 9–26 (-30) × 11–16 (-20) cm, apex acute to rounded, sometimes cuspidate, base acute to rounded, sometimes cuneate, cordate, or oblique, margin entire or wavy; adaxial side glabrous or puberulent; abaxial side puberulous, 0.1-0.4 mm long; chartaceous, drying membranaceous, brittle, light green above, paler below; veins pinnate, reticulate, secondary veins arcuate, up to twelve pairs, opposite or alternate, pale brown; abaxially pubescent and raised up to the secondary ramifications, main vein purplish on young plants. ***Inflorescences*** terminal or axillary, dendroid, to 7 cm long, pale green, puberulous; crown compact, flabellate; ***Flowers*** slightly fragrant; staminate flowers campanulate at anthesis, greenish white, 4 mm long; pistillate flowers cylindrical-campanulate at anthesis, 2 mm long. ***Infructescences*** dendroid, 10–16 cm long, drying grayish; peduncle terete, angled at base; crown lax, branches forked or pseudo-dichotomous; terminal branches with minute subulate bracteoles close to the anthocarps. ***Fruits*** anthocarps (achenes), elliptic to oblanceolate, 10–13 × 3 mm, longitudinally striate, tip cuspidate; husk softly ligneous, about 0.3 mm thick, with ten lines of viscid glands over ribs along its whole length, puberulous; glands capitate, about 0.5 mm long.

#### Habitat and ecology.

**Figure 3.**
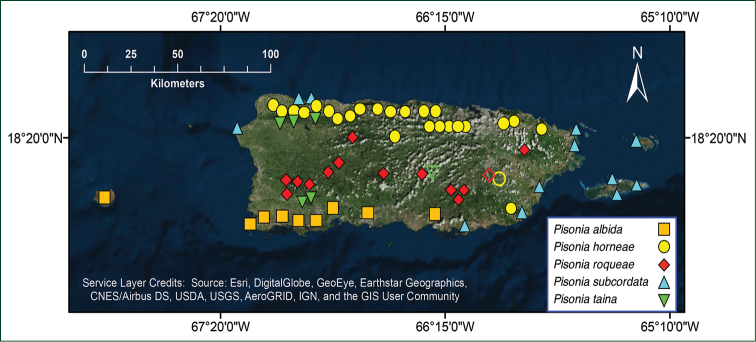
Distribution of *Pisonia* (excluding *P. aculeata*) in Puerto Rico and adjacent islands based on herbarium specimens. Empty symbols represent historical localities where populations have been presumably extirpated (see Trejo-Torres 2005 and this study for details).

*Pisonia horneae* is found in moist limestone forests at low elevations in the Northern Karst of Puerto Rico (10–150 m; Fig. 3), frequently on slopes and forest edges with relatively undisturbed vegetation. In eastern Puerto Rico, the species have been recorded from volcanic moist substrates from approximately 10 to 300 m (Fig. 3).

Although *P. horneae* is a species from low elevations and can grow near the coast, it has not been observed as part of the coastal vegetation. In contrast, the congener *P. subcordata* is distributed on coastal zones directly exposed to sea spray on elevations typically less than 10 m.

#### Phenology.

*Pisonia horneae* sheds leaves during March and April. After shedding, the plant usually produces flowers by April, though flowers have been also recorded in January. Fruits have been recorded in May and June.

#### Vernacular names.

As with other tree species of *Pisonia* from Puerto Rico, *P. horneae* is locally known as *corcho*.

#### Etymology.

We are honored to dedicate this species to Frances Elvira Worth Horne (1873–1967), an American illustrator who lived in Puerto Rico and, for 45 years, painted its plants and birds. Ms. Horne composed 750 watercolors of Puerto Rican plants to complement Nathaniel Lord Britton’s *Flora Borinqueña* (Jackson 1997), a popular book on common plants of Puerto Rico which was never published. Only 44 of her plant watercolors were published in the botanical journal *Addisonia* from 1922 to 1932 (Jackson, 1997), while 48 were included in the book Common Trees of Puerto Rico and the Virgin Islands (Little et al. 1977). She donated her collection of plant watercolors to The New York Botanical Garden in 1963 (Jackson 1997), which can be consulted at http://plants.jstor.org [accessed 02.08.2017]).

#### Abundance and conservation.

*Pisonia horneae* can be considered a rare to locally occasional species. Despite its broad distributional range along the Northern Karst of Puerto Rico, trees of *P. horneae* are usually found isolated or forming small groups. The only localities where we have observed the species as locally common include a few karstic ravines in the municipality of Quebradillas and some limestone hills with relatively undisturbed forest in Ciales (Bo. Hato Viejo) and Dorado (Bo. Rio Lajas). *Pisonia horneae* seems to be extremely rare outside the Northern Karst, as the species is only known from three scattered localities in eastern Puerto Rico in the municipalities of Rio Grande, San Lorenzo, and Yabucoa. In Rio Grande, *P. horneae* seems to be uncommon and is only known from a single specimen (described below) and an unvouchered observation of a solitary sapling made in 2006 by biologist J. A. Sustache from the SJ Herbarium (pers. comm.). The authors visited the historical locality of Cerro Gregorio in San Lorenzo in December 2016, but no *Pisonia* were located. The locality in Yabucoa contained a single individual; however, it was fruiting and multiple seedlings were observed around the tree, suggesting the presence of additional individuals at the site (see Discussion section).

Additional unvouchered localities where *P. horneae* has been reported include Arecibo (Bo. Cambalache, Cambalache State Forest, 30 m, 25 Jul 2007, MACO), Bayamón (Bo. Volcán, 45 m, 26 Dec 2007, MACO; Bo. Juan Sánchez, Parque Julio Enrique Monagas, 35 m, 13 Jan 2008, MACO) and Carolina (Bo. Hoyo Mulas, 10 m, 07 Feb 2008, MACO and N.I. Cacho).

*Pisonia horneae* is currently designated as a critical element of the flora of Puerto Rico by the Department of Natural and Environmental Resources of The Commonwealth of Puerto Rico (listed as *P. woodburyana*) (DNER 2008). It is also preliminary considered as an “Imperiled” (*sensu* NatureServe) or “Vulnerable” (*sensu* IUCN Red List) species by Gann et al. (2015-2017).

#### Additional specimens examined.

**PUERTO RICO. Arecibo**: Cambalache, 50 m, 19 Jan 1989, Liogier 36633 (NY, UPR); Bo. Dominguito, Mata de Plátano Biological Station, 93 m, 25 Jul 2003, Trejo 2349 (UPR); 142 m, 25 Jul 2003, Acevedo-Rdgz. 13404 (FTG, US); 140 m, 12 Jan 2016, Caraballo and Rivera 3380 (PAC, UPR); Finca El Tallonal, 101 m, 15 Aug 2003, Trejo 2364 (UPRRP); 100 m, 16 Mar 2004, Trejo 2585 (UPR); Bo. Miraflores, sector Biáfara, Finca Dentón, 175 m, 14 Sept 2003, Trejo 2397 (UPR); 169 m, 28 Nov 2003, Trejo 2482 (UPR); **Bayamón**: 21 Feb 1959, Woodbury s.n. (NY [#00680786], UPR [#000772]); Bo. Hato Tejas, 45 m, 21 May 2009, Caraballo et al. 2871 (UPR); **Canóvanas**: Bo. San Isidro, 10 m, 2 Jan 2017, Caraballo 3382 (PAC); **Ciales**: Bo. Hato Viejo, 160 m, 9 Jun 2007, Caraballo et al. 1818 (UPR); 130 m, 9 Jun 2007, Caraballo et al. 1822 (UPR); **Dorado**: Bo. Higuillar, 8 Feb 1964, Liogier 10670 (F, MAPR, NY); Bo. Sabana, northeast of Regadera, off Road 691, 5-10 m, 12 May 1985, Proctor 41173 (UPR); Bo. Río Lajas, 15 m, 20 Jun 2003, Trejo and Caraballo 2279 (UPR); **Hatillo**: Bo. Capáez, ca. 5 m, 12 Jan 2006, Axelrod 13357 (UPRRP); **Isabela**: Quebrada La Sequia, 75 m, 2 Oct 2005, Caraballo et al. 639 (UPR); Guajataca gorge, 75 m, 26 Jan 2006, Caraballo et al. 1425 (UPR); Cordillera Jaicoa, 200 m, 23 Aug 2006, Caraballo 1196 (UPR); **Loíza**: Bo. Piñones, Piñones Forest Reverse, 13 Mar 1980, Del Llano s.n. (UPRRP [#96]); **Manatí**: Bo. Tierras Nuevas Saliente, 03 May 2001, Axelrod and Zachariades 11754 (UPRRP); Bo. Tierras Nuevas Poniente, Hacienda La Esperanza, 10 m, 14 Mar 2006, Caraballo et al. 919 (UPR); 12 m, 28 Mar 2006, Caraballo and Rivera 976 (UPR); **Moca**: Bo. Aceitunas, 175 m, 1 Mar 2008, Caraballo 2455 (UPR); **Quebradillas**: Bo. San José, Quebrada Bellaca, 52 m, 14 Oct 2003, Trejo and Caraballo 2444 (UPR); Bo. Cacao-Terranova, Quebrada Las Talas [El Gallo], 85 m, 21 Aug 2004, Trejo and Caraballo 2751 (UPR); Bo. Cocos, Quebrada Bellaca, 101 m, 9 Mar 2005, Trejo et al. 2873 (NY, UPR); limestone hills, ca. 150 m, 22 May 2005, Axelrod et al. 13062 (UPRRP); **Río Grande**: Rio Mar, 100 m, 5 Aug 1988, Liogier and Martorell 36637 (NY, UPR); **San Lorenzo**: Prope Hato Grande in sylva primaera montis Gregorio, 31 Aug 1885, Sintenis 2705 [mixed specimens, see Introduction and additional specimens examined for *P. roqueae*] (B [10-0217019]; GH [00037445]; K [K000036122]); **Toa Baja**: Bo. Candelaria, E sector, Mogotes de Nevárez, 20-90 m, 27 Mar 1989, Proctor 45450 (US); 5 Mar 1914, Britton et al. 1515 (NY); Campanillas, 8 Jan 1959, Woodbury s.n. (UPR [#027151]); Nevárez, 75 m, 16 Mar 2006, Caraballo et al. 931 (UPR); **Vega Alta**: Bo. Espinosa, 11 Feb 1926, Gleason and Cook A134 (NY); Bo. Sabana, 5–10 m, 12 May 1985, Proctor 41173 (IJ); hill NE of Regadera, 5 m, 1 Apr 1987, Proctor 43284 (US); Bo. Regadera, Axelrod 84 (UPRRP); **Yabucoa**: Cuchilla de Pandura, 175 m, 13 June 2006, Caraballo and Flecha 1088 (UPR); **Without locality**: without locality, 8 Nov 1886, Sintenis 4355 [see Introduction and additional specimens examined for *P. roqueae*] (B, n.v., probably lost).

### Pisonia roqueae

Taxon classificationPlantaeCaryophyllalesNyctaginaceae

Trejo & Caraballo sp. nov.

urn:lsid:ipni.org:names:77165903-1

Figs 3 , 4 , 5 , 6

Pisonia subcordata sensu Heimerl and Urban (1905), not Swartz (1788).
Pisonia subcordata Swartz var. typica Heimerl f. gigantophylla Heimerl. Bot. Jahrb. Syst. 21: 630. 1896. Pro parte (see Additional specimens examined section). Lectotype (designated here): Barranquitas: Prope Barranquitas in sylva primaera montis Torrecilla, 30 Oct 1885, Sintenis 2141 (GH [00037444]; K [H2005/00995]).
“Pisonia borinquena Proctor”, *nomen nudum*.

#### Type.

PUERTO RICO. Municipio de San Germán, Bo. Caín Alto, Maricao State Forest, in a moist ravine, south of Road 120 km 15.6, 850 m, 23 June 1992, G.R. Proctor, R. Padrón, and R. Rivera 48064 (holotype: US! [#00707324]; isotype: MO! [#04580139]).

#### Diagnosis.

*Pisonia roqueae* is distinguished from congeners from Puerto Rico by a combination of the following characters: staminate inflorescences with globose crowns, twigs glabrescent, leaves coriaceous and glabrescent, and fruits clavate and reddish-black with five rows of viscid glands on their distal half.

#### Description.

***Trees*** dioecious up to 25 m high with trunks up to 1 m in diameter. ***Bark*** finely and vertically striated, grayish with lenticels about 3 mm in diameter. ***Twigs*** ancipitous, ferruginous-brownish, and pubescent when young; terete, glabrescent, and grayish when old. ***Leaves*** clustered towards the ends of branches, opposite, subopposite, or sub-verticillate, decussate; petioles up to 3.5 cm long, yellowish green; blades elliptic, obovate, or roundish, 10–17 (-25) cm × 7–12 (-16) cm, apex acute to rounded, sometimes cuspidate, base acute to cuneate, margin entire or slightly wavy, adaxial and abaxial sides glabrous, coriaceous, drying coriaceous, dark green above, slightly paler below; veins pinnate, reticulate, up to eleven pairs, opposite or alternate, blackish. ***Inflorescences*** terminal or axillary, dendroid, to 7 cm long; crowns compact; staminate inflorescences with a globose crown; pistillate inflorescences with a flabellate crown. ***Flowers*** fragrant; pistillate flowers with perianth cylindrical-campanulate-oblong at anthesis, 2 mm long, puberulent; staminate flowers perianth campanulate at anthesis, green, about 3 mm long, puberulent. ***Infructescences*** dendroid, 4-7 cm long, drying reddish or brownish; peduncle angled, 2-3 cm long; branches irregularly forked, with minute bracteoles around the base of anthocarps. ***Fruits*** anthocarps (achenes), clavate, 10-15 mm x 2 mm, longitudinally striate, tip cuspidate; husk glabrous, with five lines of glands over discrete ribs along the distal third or half; glands capitate, about 0.5 mm long, viscid.

#### Habitat and ecology.

*Pisonia roqueae* is mainly distributed from middle to high elevations. In the Central Mountain Range and Sierra de Luquillo, the species have been found on wet and moist serpentine or volcanic forests from 480 to 950 m. The only known locality from the Northern Karst is on a wet limestone forest at ca. 330 m.

Unlike other species of *Pisonia* from Puerto Rico, adult trees of *P. roqueae* can reach considerable heights (> 15 m). Some large trees can be found at the Maricao State Forest and at the Luquillo Mountains in EL Verde Field Station, including the Luquillo Forest Dynamics Plot (http://luq.lternet.edu), where the species (treated as either “*P. borinquena*” or *P. subcordata*) has been included in long-term studies (e.g., Kress et al. 2010).

Some species of *Pisonia* from Puerto Rico, including *P. roqueae*, are common hosts for native species of mistletoes. For example, *P. albida* and *P. roqueae* are important hosts for *Phoradendron anceps* (Spreng.) M. Gómez (Santalaceae) at the Guánica State Forest and the Maricao State Forest, respectively (Pinto 2005). Similarly, *P. subcordata* has been reported as host for the mistletoes *Dendropemon caribaeus* Krug & Urb. (Loranthaceae) and *P. anceps* in northern Puerto Rico (M.A. Vives-Heyliger, pers. comm.).

#### Phenology.

*Pisonia roqueae* has been recorded flowering in April, June, and July, and fruiting from January to April.

#### Vernacular names.

As with other tree species of *Pisonia* from Puerto Rico, *P. roqueae* is locally known as *corcho* or *corcho blanco*.

#### Etymology.

It is our honor to name *Pisonia roqueae* after Dr. Ana Cristina Roqué Geigel de Duprey (1853–1933), an amateur ethnobotanist from Puerto Rico who dedicated over three decades of her life to prepare the bilingual manuscript “*Botánica Antillana: Introduction to the study of the picturesque flora of Porto Rico and West Indies*”, aimed to make botany accessible to the general public. Her manuscript (Roqué de Duprey 1925) was never published and remained in oblivion to the botanical community until recently when JCTT and collaborators divulged its existence (Martínez 2015). Roqué de Duprey is mostly known for being an educator, writer, suffragist, and one of the founders of the University of Puerto Rico-Mayagüez, among other educational institutions.

#### Abundance and conservation.

*Pisonia roqueae* has been observed as a locally common tree in two localities in the eastern and western Central Mountain Range (Monte La Torrecilla in Barranquitas and Maricao State Forest, respectively), occasional at the El Yunque National Forest (Luquillo Mountains), and rare elsewhere. It has been also recorded from Cayey (including an unvouchered locality at Bo. Jájome, 300–350 m, 18 Feb 2015, by O. Monzón), Guayama at Sierra de Cayey, and a single record from the Northern Karst at Sabana Hoyos, Arecibo. The species is preliminary considered as “Imperiled” (*sensu* NatureServe) or “Vulnerable” (*sensu* IUCN Red List) by Gann et al. (2015–2017).

#### Additional specimens examined.

**PUERTO RICO. Arecibo**: Bo. Sabana Hoyos, Finca Las Abras, 330 m, 9 Sept 2002, Trejo 1773 (UPRRP); 300 m, 10 Jul 2005, Trejo 2996 (UPR); **Barranquitas**: Bo. Barrancas, Monte La Torrecilla, 900-1100 m, 19-20 Mar 1915, Britton et al. 5628 (NY); 950 m, 1 Apr 1989, Proctor 45465 (FTG, UPR); 930 m, 24 Jul 2003, Trejo 2340 (FLAS, GH, UPR); Trejo 2342 (NY, UPR); Trejo 2343 (UPR); 5 Mar 2004, Trejo 2552 (UPR); Trejo 2553 (UPR); **Cayey**: Cercadillo, 600 m, 10 Feb 1983, Liogier 33875 (UPR); **Ciales**: Bo. Toro Negro, Tres Picachos, 700 m, 29 Jul 1993, Axelrod 6694 (JBSD, UPRRP); Bo. Cialitos, Los Tres Picachos, 675 m, 4 Apr 2008, Caraballo et al. 2561 (UPR); **Guayama**: Bo. Carmen, 290 m, 4 Jun 2016, Areces et al. 1140 (UPRRP); **Maricao**: Maricao Forest, 800 m, 26 Jun 1938, Sargent 506 (MO); 500 m, 2 Oct 1938, Sargent 633 (MO); Vivero de Peces, 850 m, 13 Jul 1950, Little 13380 (F, NY, UPR); 880 m, 15 Jul 1950, Little 13404 (F, NY, UPR); 26 Nov–4 Dec 1963, Duke 7134 (MO); Monte Alegrillo, 890-900 m, 5 Apr 1987, Proctor 43322 (US); Monte del Estado, 700 m, 6 May 1990, Caminero 282 (MAPR); Bo. Maricao Afuera, Monte del Estado, 850 m, 15 May 1991, Caminero 443 (MAPR); 480 m, 3 Apr 1996, Cedeño 881 (MAPR); 550 m, 4 Apr 1996, Cedeño 904 (MAPR); 840 m, 29 Feb 2004, Carlo 21 (UPR); 875 m, 27 May 2009, Caraballo et al. 2878 (UPR); s.c. s.n. (MAPR [016698]); **Sabana Grande**: Bo. Sabana-Tabonuco, 750 m, 5 Mar 2004, Trejo 2648 (UPR); **San Germán**: Bo. Caín Alto, Maricao Forest, 810–830 m, 23 Jan 1988, Proctor 44432 (US); **San Lorenzo**: Prope Hato Grande in sylva primaera montis Gregorio, 31 Aug 1885, Sintenis 2705 [mixed specimens, see Introduction and Additional specimens examined for *P. horneae*] (B [10-0217019]; GH [00037445]; K [K000036122]); **Río Grande**: El Verde Experimental Station, 29 Jun 1963, Smith 345 (EVFS [El Verde Field Station herbarium]); August 1963, Smith 470 (EVFS); 15 May 1964, Smith 1122 (EVFS); LTER plot, 350 m, 18 Jun 1990, Moestl 9 (EVFS); 419 m, 2 Mar 2013, Areces 748 (UPRRP); Río Sonadora, 350 m, 18 Jun 1991, Taylor 10453 (MO); 19 May 1994, Taylor 11689 (MO); **Utuado**: without locality, 1916, Strube s.n. “A” (MO [#653326]); Bo. Roncador, Hacienda Verde, 514 m, 8 Aug 2004, Trejo 2731 (UPR); **Without locality**: without date, 8 Nov 1886, Sintenis 4355 [see Introduction and Additional specimens examined for *P. horneae*] (B, n.v., probably lost).

## Discussion

**Figure 4.**
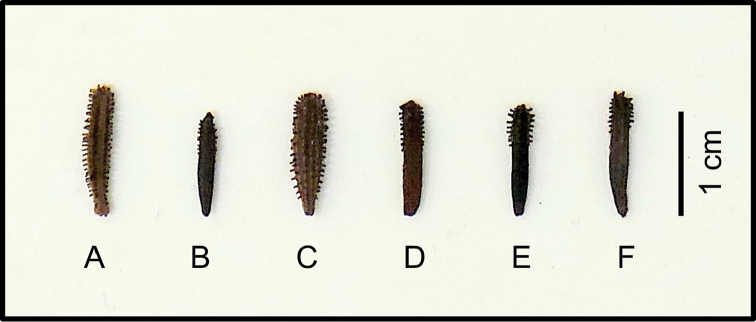
Fruits of the six species of *Pisonia* from Puerto Rico. **A** *P. aculeata* **B** *P. albida* **C** *P. horneae* **D** *P. roqueae* **E** *P. subcordata* **F** *P. taina*. Reference specimens: **A** Rose 3548, US **B** Little 13219, US **C** Acevedo-Rdgz. 13404, US **D** Proctor 44432, US **E** Breckon 7766, US; and **F** Trejo 2371, US.

With the formal addition of *P. horneae* and *P. roqueae*, the genus *Pisonia* in Puerto Rico is represented by six species, including three Puerto Rican endemics, two West Indian-restricted, and one pantropical. The distribution of the six species of *Pisonia* from Puerto Rico seems to follow a geographic and environmental pattern (Fig. 4): the vine *P. aculeata* L., the most widespread species in the genus with a worldwide distribution, is found at lower and middle elevations across the island; *P. albida*, a tree from Hispaniola and Puerto Rico, is distributed along dry coastal forests in southern lowlands; *P. horneae* is distributed in the moist limestone forests of the Northern Karst and the eastern Central Mountain Range; *P. roqueae* is a species restricted to wet forests with elevations above 300 m along the Central Mountain Range and the Luquillo Mountains; *P. subcordata*, a tree distributed throughout the West Indies, is mainly found on coastal forests and thickets along the northern and eastern coast; and *P. taina* Trejo, an endemic and rare tree from Puerto Rico, has a scattered distribution across the central and western parts of the island (Trejo-Torres 2005). The distributional ranges of *P. albida*, *P. horneae*, *P. roqueae*, and *P. subcordata* seems to be consistent with specific substrate types and/or environmental conditions. However, we still lack scientific studies exploring possible biotic or abiotic factors shaping these distributions. A possible explanation is the existence of species-specific interactions with ectomycorrhizal fungi, which can help species survive in particular abiotic conditions (Van der Putten et al. 2010, Pickles et al. 2015) and have been found to interact with *Pisonia* and other members of the Pisonieae clade for at least 14 Myr (Hayward and Horton 2014). We also lack molecular studies to infer the phylogenetic relationships and species divergence times among Caribbean *Pisonia*.

The most reliable characteristics to set apart the six species of *Pisonia* from Puerto Rico are the morphology of inflorescences, flowers, and fruits along with leaf shape and size. However, *Pisonia* trees have a fugacious reproductive season and herbarium specimens are often found either sterile or fertile but lacking well-developed leaves (Fig. 6). As a result, many specimens are incomplete representations of the species, which has partially contributed to their misidentification (Trejo-Torres 2005). Therefore, when collecting *Pisonia* in the field we recommend to include, whenever possible, multiple vouchers from a single plant or population throughout seasons to properly document the range of phenological stages of plants, including fertile and sterile material. *Pisonia* fruits usually persist on branches or on the ground below trees during several months. Hence, we included a comparison of fruit morphology for the six species of *Pisonia* from Puerto Rico to assist with their identification (Fig. 4). Observational information may also assist with identification of the two-new species here described and we have noted that, unlike other *Pisonia* from Puerto Rico, fresh leaves of *P. horneae* are usually light green and young plants often have a purplish midvein. Likewise, leaves of *P. roqueae* are typically dark green and shiny.

**Figure 5.**
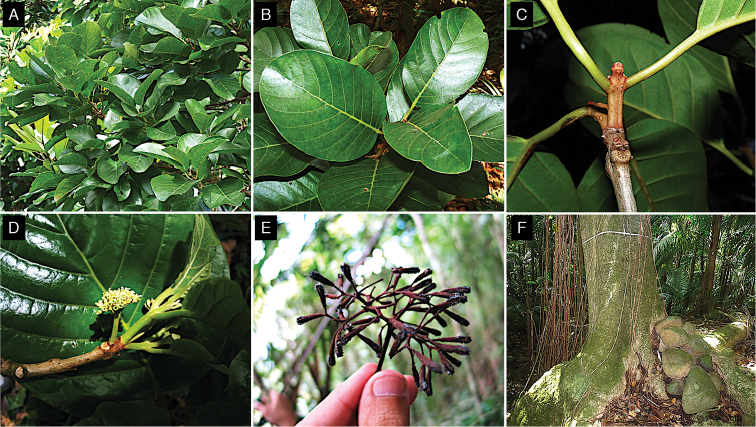
*Pisonia roqueae*. **A** Foliage **B** Adaxial side of mature leaves **C** Twig with ferrugineous-brownish pubescence, turning grayish as it ages **D** Pistillate inflorescence and young leaves **E** Ripe infructescence **F** Trunk of an adult tree. Photo credits: **A–C, F**: Fabiola Areces (Areces Berazain et al. 2013-2017); **D–E**: JCTT.

**Figure 6.**
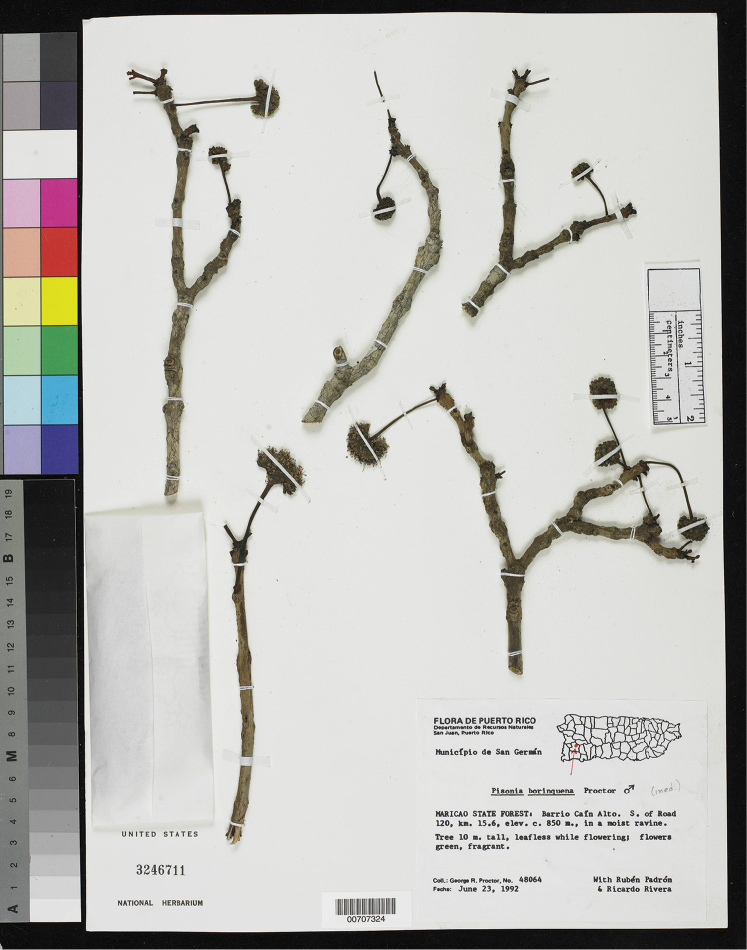
Holotype specimen of *Pisonia roqueae* from the National Herbarium of the Smithsonian Institution (Proctor et al. 48064, US [00707324]). Note the dendroid staminate inflorescences with globose crowns and the leafless branches during the flowering period. Photo courtesy of The United States National Herbarium, Smithsonian Institution.

### Notes on reproduction and conservation

Most of the observed plants of *P. horneae* and *P. roqueae* were either isolated or forming small groups. Although both species have broad distributions within their respective habitats, most trees are restricted to ravine banks, cliffs, or rocky areas, especially for *P. horneae*. These observations might suggest that either trees tend to colonize these particular habitats due to physiological requirements, or that they represent relicts of a former, more continuous population that was severely fragmented during the intense deforestation period experienced in the island for the past centuries.

As with other *Pisonia* species, both *P. horneae* and *P. roqueae* are dioecious and their flowers might require pollen to set fruits (Douglas and Manos 2007, Nores et al. 2015). However, at least two species of *Pisonia* from Puerto Rico (*P. taina* (Trejo-Torres 2005) and *P. subcordata* (M.A. Vives-Heyliger, pers. comm.)) have been reported as rarely subandroecious, a condition that can help mitigate the impact of reproductive isolation by inbreeding. We currently lack information on reproductive biology for the vast majority of *Pisonia* species (Douglas and Manos 2007), but field observations made by one of the authors suggest that *P. horneae* and *P. subcordata* can achieve high fruit sets in sites where trees are locally abundant (MACO, pers. obs.). This suggests that *Pisonia*, as other dioecious tropical trees, can be particularly susceptible to reproductive failure from isolation and low population sizes (House 1992). Reproductive success of dioecious plants will depend on multiple factors such as the spatial distribution of trees, the male-female ratio within populations, and the foraging range of their effective pollinators (Lin et al. 2015). Thus, conservation efforts, besides protecting extant trees and natural areas where the species are present, should take into consideration increasing neighborhood densities by planting additional individuals near isolated trees, even though these actions would not warrant maintenance of genetic diversity and the long-term survival of the species.

To our knowledge, there are no reports of seed dispersal for any Neotropical *Pisonia*, which contrast with the Indo-Pacific region where seed dispersal has been well-documented, especially for *P. grandis* R. Br. and *P. umbellifera* (J.R. Forst. & G. Forst.) Seem. (e.g., Ridley 1930, Cleland 1952, Walker et al. 1991, Murphy and Legge 2003, Burger 2005). In 2005 after an intense fruiting season at one of the densest known populations of *P. horneae* at the municipality of Quebradillas in northwestern Puerto Rico, infructescences detached from branches when fruits were ripe, but they remained under their maternal trees. Subsequently, seedlings grew densely clustered and the vast majority of them died within the next few months (Fig. 1D; MACO, pers. obs.). These observations, along with the low recruitment detected beyond parental trees, suggest that at least some populations of *P. horneae* might be experiencing low seed dispersal rates.

### Key to the species of *Pisonia* from Puerto Rico

**Table:** 

1	Woody liana; twigs armed with spines	***P. aculeata***
–	Shrubs or trees; twigs unarmed	**2**
2	Leaf veins at abaxial side raised up to the finer ramifications	***P. taina***
–	Leaf veins at abaxial side not raised up to the finer ramifications	**3**
3	Twigs glabrescent (except at leaf axils); dried leaves coriaceous; fruits drying black or reddish black	**4**
–	Twigs puberulous; dried leaves chartaceous or membranaceous; fruits drying brown or gray	**5**
4	Leaves puberulent, especially on the abaxial side and along veins; fruits ca. 1.3 cm long × 2.5 mm wide in average; staminate inflorescences with a globose crown	***P. roqueae***
–	Leaves glabrous, sometimes slightly puberulent on the abaxial side at base of main vein; fruits ca.1 cm long × 1.5 mm wide in average; staminate inflorescences with a flabellate crown	***P. subcordata***
5	Leaves up to 14 cm long, usually with a constant elliptic shape, chartaceous when dried, and whitish on the abaxial side; fruits brown, clavate with five rows of viscid glands along the distal end	***P. albida***
–	Leaves up to 40 cm long, variable in shape, membranaceous when dried, and greenish on the abaxial side; fruits gray, ellipsoid with ten rows of viscid glands along the whole fruit	***P. horneae***

## Supplementary Material

XML Treatment for Pisonia horneae

XML Treatment for Pisonia roqueae

## Exsiccatae

Acevedo-Rdgz., P.A. 13404 (horneae)

Areces, F. 748, 1140 (roqueae)

Axelrod, F.S. 84 (horneae); 6694 (roqueae); 11754, 13062, 13357 (horneae)

Britton, N.L. 1515 (horneae); 5628 (roqueae)

Caminero, G. 282, 443 (roqueae)

Caraballo, M.A. 639, 919, 931, 976, 1088, 1196, 1425, 1818, 1822, 2455 (horneae); 2561 (roqueae); 2871 (horneae); 2878 (roqueae); 3380, 3382 (horneae)

Carlo, T.A. 21 (roqueae)

Cedeño, J.A. 881, 904 (roqueae)

Duke, J.A. 7134 (roqueae)

Gleason, H.A. A134 (horneae)

Liogier, H.A. 10670 (horneae); 33875 (roqueae); 36633, 36637 (horneae)

Little, E.L. 13380, 13404 (roqueae)

Moestl, S. 9 (roqueae)

Proctor, G.R. 41173, 43284 (horneae); 43322, 44432 (roqueae); 45450 (horneae); 45465, 48064 (roqueae)

Taylor, C.M. 10453, 11689 (roqueae)

Trejo, J.C. 1773 (roqueae); 2279, 2310 (hornena); 2340, 2342, 2343 (roqueae); 2349, 2364, 2397, 2444, 2482 (horneae); 2552, 2553 (roqueae); 2585 (horneae); 2648, 2731 (roqueae); 2751, 2873 (horneae); 2996 (roqueae)

Sargent, F.H. 506, 633 (roqueae)

Sintenis, P.E.E. 2141 (roqueae); 2705 (horneae and roqueae)

Smith, R.F. 345, 470, 1122 (roqueae)

Strube, L.B. s.n. “A” (roqueae)
